# Comparative Aspects of Structure and Function of Cnidarian Neuropeptides

**DOI:** 10.3389/fendo.2020.00339

**Published:** 2020-05-27

**Authors:** Toshio Takahashi

**Affiliations:** Suntory Foundation for Life Sciences, Bioorganic Research Institute, Kyoto, Japan

**Keywords:** *Hydra*, Cnidaria, neuropeptide, metamorphosis, myoactivity, interstitial stem cell, neuron differentiation

## Abstract

Cnidarians are early-branching animals in the eukaryotic tree of life. The phylum Cnidaria are divided into five classes: Scyphozoa (true jellyfish), Cubozoa (box jellyfish), Hydrozoa (species, *Hydra* and *Hydractinia*), Anthozoa (sea anemone, corals, and sea pen), and Staurozoa (stalked jellyfish). Peptides play important roles as signaling molecules in development and differentiation in cnidaria. For example, cnidaria use peptides for cell-to cell communication. Recent discoveries show that *Hydra* neuropeptides control several biological processes including muscle contraction, neuron differentiation, and metamorphosis. Here, I describe the structure and functions of neuropeptides in *Hydra* and other cnidarian species. I also discuss that so-called primitive nervous system of *Hydra* is in more complex than generally believed. I also discuss how cnidaria use peptides for communication among cells rather than in higher animals.

## Introduction

Molecular phylogenetic studies show that Cnidaria are the sister group of Bilateria. Ancestral Cnidarians diverged over 500 million years ago in animal evolution. Despite the long course of evolution, the nervous systems of cnidarians are differentiated (1). Cnidarian species are also mainly classified into two groups according to the unique life cycle, the anthozoans and medusozoans (1). Anthozoa lives exclusively as polyps. Among medusozoans, Cubozoa and Scyphozoa predominantly live as medusae. On the other hand, Hydrozoa usually follows a life cycle where the species alternate between these two forms except for *Hydra* and *Hydractinia*. Staurozoa lives exclusively as polyps.

Cnidaria such as *Hydra* are composed of multiple cell types that represent the fundamental architecture of multicellular organisms. *Hydra* exhibits a simple body plan with a head and tentacles on one end and a foot on the opposite end of a hypostome. The gastric region is located between the head and foot. The body is composed of two layers, ectoderm and endoderm, which are separated by an extracellular matrix, the mesoglea. The cells of both epithelial layers also function as muscle cells. *Hydra* also have multipotent interstitial stem cells, which differentiate into nerve cells (2), nematocytes (2), gland cells (3), and germ cells (4). *Hydra* as a member of cnidaria represents an attractive model to understand axial pattern formation into head- and foot-specific tissues.

The nervous system of *Hydra* is simple and is composed of a nerve net that extends throughout the animal. The cnidarian nervous system is mainly peptidergic (5). Classical molecules such as acetylcholine also contribute to the *Hydra* nervous system (6).

**Figure 1:**
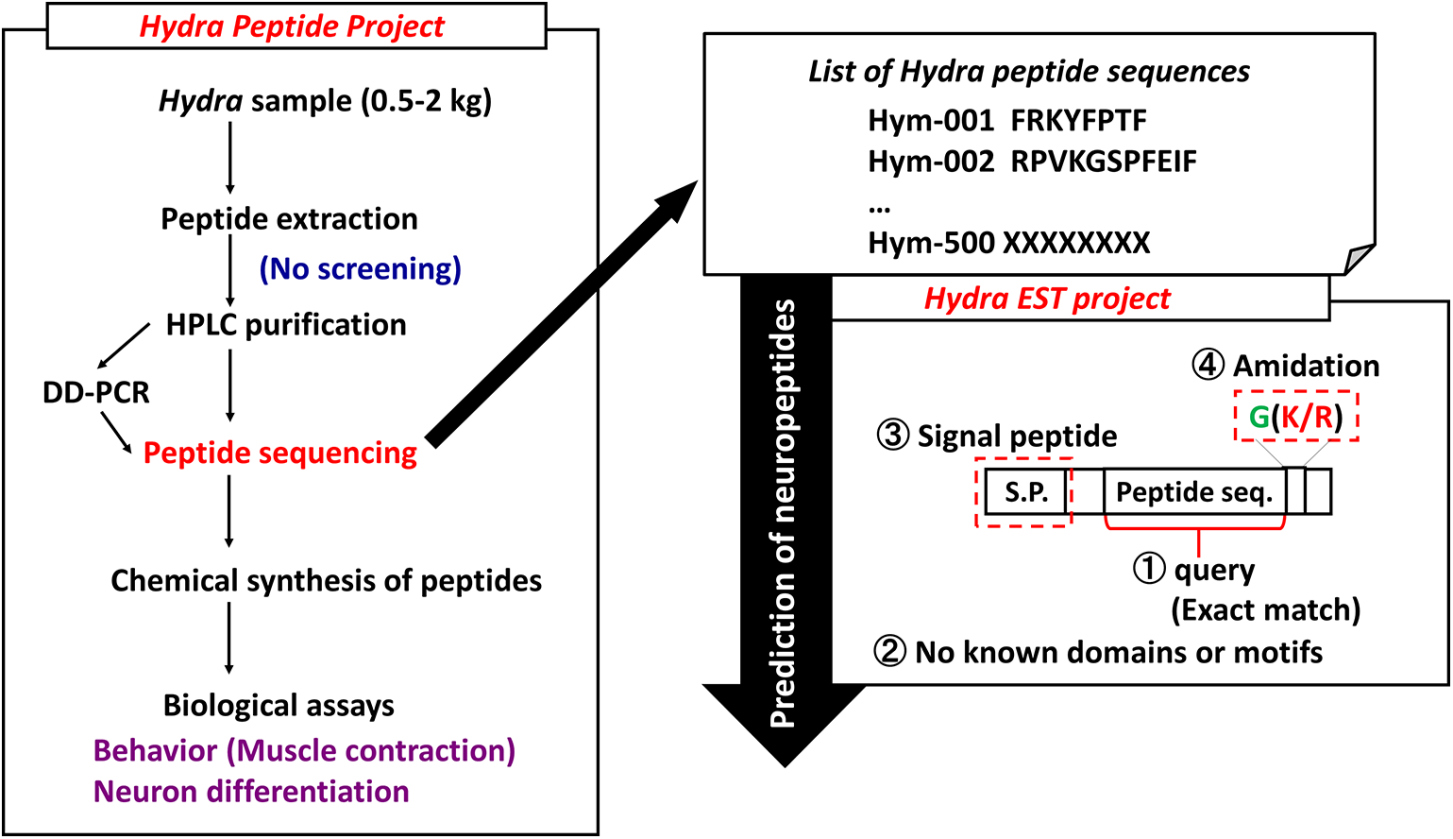
Strategy to identify *Hydra* neuropeptides. DD-PCR, differential display PCR; HPLC, high performance liquid chromatography.

Peptides play important roles as hormones and neurotransmitters and they are involved in the maintenance of a variety of developmental stages. However, little is known about whether they are involved in differentiation and development. In *Hydra*, theoretical model suggests that small molecules such as peptides are transported to establish morphogenetic gradients that regulate patterning processes. To systematically identify and characterize peptide signaling molecules, we started the Hydra Peptide Project (7). By using the strategy illustrated in Figure 1, many peptides were extracted and purified with successive steps of high performance liquid chromatography (HPLC). Signaling peptides were identified by their effect on the gene expression profile of *Hydra* by using differential display (DD)-PCR. Positive peptides were chemically synthesized, the synthetic peptides were used for biological assays including behavioral (muscle contraction), neuron differentiation, and others. Furthermore, introduction of the *Hydra* Expressed Sequence Tag (EST) Project has enabled us to identify transcripts for novel peptides even more efficiently (Figure 1) (8).

The primary aim of the present review is to describe the structures and functions of peptide signaling molecules such as neuropeptides in cnidarians, especially in *Hydra*.

## Cnidarian Neuropeptides

### FMRFamide-Like Peptides (FLPs)

The peptide FMRFamide was originally purified from the cerebral ganglion of the clam *Macrocallista nimbosa* (9, 10). Other mollusks and members of most other phyla express peptides with a similar sequence. FMRFamides are categorized into two groups depending on the structural similarity with FMRFamide. The first category consists of FMRFamide-related peptides (FaRPs), which include encode for multiple peptides with the C-terminal FMRFamide or FLRFamide (11). The second category of FMRFamides includes FLPs, which are peptides that have only the RFamide sequence at C-termini (12). Therefore, FaRPs and all other RFamide peptides are considered FLPs. Krajniak (13) excellently reviewed FaRPs in invertebrates. This overview primarily focuses on cnidarian FLPs.

**Figure 2:**
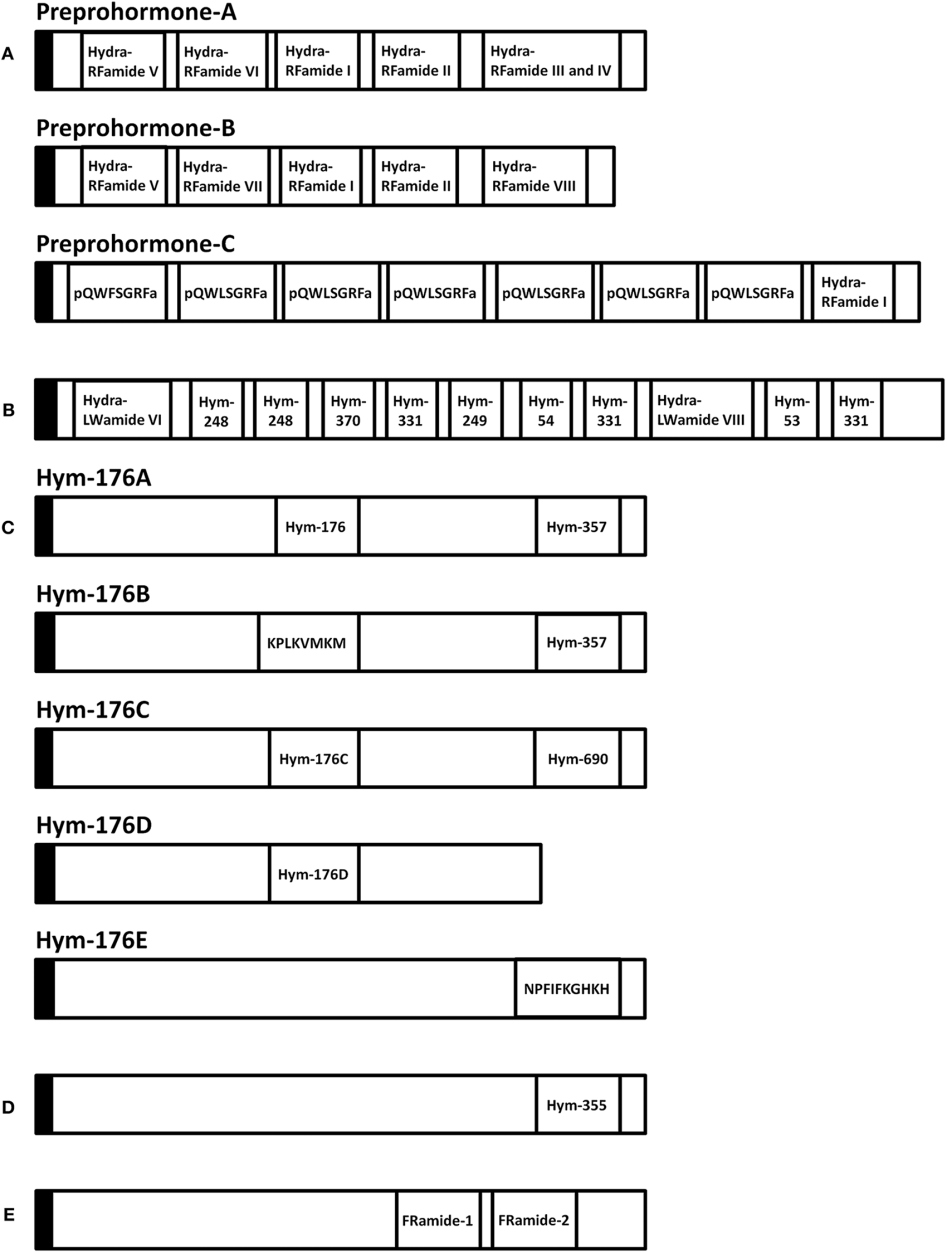
Schematic representation of eleven preprohormones in *Hydra*. **(A)** Preprohormone-A contains unprocessed Hydra-RFamide I, II, III, IV, V, and VI. Preprohormone-B contains unprocessed Hydra-RFamide I, II, V, VII, and VIII. Preprohormone-C contains Hydra-RFamide I and two putative neuropeptide sequences (pQWFSGRFa and pQWLSGRFa). **(B)** The GLWamide precursor contains unprocessed GLWamides (Hym-53, 54, 248, 249, 331, and 370) and two putative neuropeptide sequences (Hydra-LWamide VI and VIII). **(C)** The Hym-176 precursor (Hym-176A) contains one copy of unprocessed Hym-176 and Hym-357. Hym-176B contains Hym-357 and one putative neuropeptide (KPLKVMKM). Hym-176C and Hym-176D contain one copy of Hm-176-homologous peptide (Hym-176C and Hym-176D), respectively. Hym-176C also contains one unprocessed Hym-690. Hym-176E contains one putative neuropeptide sequence (NPFIFKGHKH). **(D)** The Hym-355 precursor contains one unprocessed Hym-355. **(E)** The FRamide precursor contains unprocessed FRamide-1 and−2. Black box: signal sequence, a: amide.

A variety of FLPs are expressed in the evolutionarily ancient nervous system of cnidarians (Table 1). Peptides with GRFamide at the C-terminus have been found in a scyphozoan (the jellyfish *Cyanea lamarckii*) (15), three hydrozoans (*Hydra magnipapillata*, the hydromedusa *Polyorchis penicillatus*, and *Hydractinia echinata*) (16–21), and an anthozoan (the sea anemone *Anthopleura elegantissima*) (14), whereas peptides with TRFamide and/or RRFamide at the C-terminus have been described in another anthozoan (the sea anemone *Nematostella vectensis*) (22). All mature neuropeptides are controlled by highly regulated secretion pathways. Usually, a precursor of a neuropeptide is incorporated as a preprohormone in the endoplasmic reticulum, where it is converted into a prohormone. Next, prohormones move to the Golgi apparatus for endoproteolysis and/or amidation at the C-terminus, which results in the final active peptide. FLPs have been identified in numerous cnidarians. A *Calliactis parasitica* cDNA includes 19 copies of Antho-RFamide (Table 1), two copies of FQGRFamide, and one copy of YVPGRYamide (24). Two cDNAs have been isolated from *Anthopleura elegantissima*; one cDNA includes 13 copies of Antho-RFamide (Table 1) and nine other FLPs; the second cDNA includes 14 copies of Antho-RFamide and eight other FLPs (25). *Renilla koellikeri* has 36 copies of Antho-RFamide (26). A *Polyorchis penicillatus* cDNA includes one copy of Pol-RFamide I (Table 1) and 11 copies of Pol-RFamide II (Table 1), in addition to another predicted FLP (27). In *Hydra*, RFamides are spliced from three different preprohormones called A, B, and C (Figure 2A). Preprohormone-A includes six Hydra-RFamides (Hydra-RFamide I-VI) (Figure 2A) (Table 1) (19). Preprohormone-B has one copy of Hydra-RFamide I and Hydra-RFamide II and three probable Hydra-RFamides (Hydra-RFamide V, VII, and VIII) (Figure 2A) (19). Preprohormone-C has one copy of Hydra-RFamide I and seven copies of additional neuropeptide sequences (one copy of pQWFSGRFamide and six copies of pQWLSGRFamide) (Figure 2A) (19). In *Hydractinia echinata*, one copy of He-RFamide is present (Table 1) (21). In *Nematostella vectensis*, three FLPs [Nv-RFamide I and II and RFamide (ID:17)] are present (Table 1) (22, 23). Collectively, precursor-encoding cnidarian FLP cDNAs yield many neuropeptides with great structural diversity, indicating that they have great functional diversity as well.

**Table 1:** FLPs in cnidarians.

**Name**	**Peptide sequence**	**Species**	**Reference**
Antho-RFamide	pQGRFamide	Anthopleura elegantissima	(14)
Cyanea-RFamide I	pQWLRGRFamide	Cyanea lamarckii	(15)
Cyanea-RFamide II	pQPLWSGRFamide		
Cyanea-RFamide III	GRFamide		
Pol-RFamide I	pQLLGGRFamide	Polyorchis penicillatus	(16)
Pol-RFamide II	pQWLKGRFamide		(17)
Hydra-RFamide I	pQWLGGRFamide	Hydra magnipapillata	(18)
Hydra-RFamide II	pQWFNGRFamide		
Hydra-RFamide III	KPHLRGRFamide		
Hydra-RFamide IV	HLRGRFamide		
Hydra-RFamide V	pQLMSGRFamide	Hydra magnipapillata	(19)
Hydra-RFamide VI	pQLMRGRFamide		
Hydra-RFamide VII	pQLLRGRFamide		
Hydra-RFamide VIII	KPHYRGRFamide		
Hydra-RFamide IX	HYRGRFamide		
Hydra-RFamide X	KPHLIGRFamide	Hydra magnipapillata	(20)
Hydra-RFamide XI	pQLMTGRFamide		
He-RFamide	pQWLKGRFamide	Hydractinia echinata	(21)
Nv-RFamide I	pQITRFamide	Nematostella vectensis	(22)
Nv-RFamide II	VVPRRFamide		
RFamide (ID:17)	pQGRFGREDQGRFamide		(23)

*pQ, pyroglutamate*.

Cnidarian FLPs control several functions, such as muscle contraction, feeding, sensation, reproduction, metamorphosis, and movement of larvae. Treatment of the sea anemone *Calliactis parasitica* with Antho-RFamide increases muscle tone, contraction amplitude, and contraction of slow muscles (28). In individual autozooid polyps of *Renilla koellikeri*, Antho-RFamide also leads to tonic contractions in the rachis and peduncle (29). In *Hydra*, Hydra-RFamide III mediates pumping of the peduncle in a dose-dependent manner (30).

FMRFamide activates a Na^+^ channel identified in snails (31, 32). Three cation channel subunits of the degenerin (DEG)/epithelial Na^+^ channel (ENaC) gene family were cloned from the freshwater polyp *Hydra magnipapillata* and designated *Hydra* Na^+^ channel (HyNaC)2–4 (33). Subsequently, a novel subunit, designated HyNaC5, was cloned, and expression of the gene was shown to be co-localized with HyNaC2 and HyNaC3 at the base of the tentacles (34). Co-injection of HyNaC5 with HyNaC2 and HyNaC3 genes in *Xenopus* oocytes strongly enhances the current amplitude after peptide application and increases the affinity of the channel for Hydra-RFamide I and II (34). HyNaC2/3/5 is assembled into a functional heterotrimeric channel that is activated by Hydra-RFamide I with high affinity. The experimental data of HyNaCs suggested that secretion of Hydra-RFamide I and/or II induces tentacle contraction, perhaps during feeding (33, 34). Seven additional HyNaC subunits, HyNaC6-HyNaC12, were cloned, and all belong to the DEG/ENaC gene family (35). These subunits and the four originally identified subunits self-assemble in *Xenopus* oocytes to create 13 different ion channels that show high-affinity binding of Hydra-RFamide I and II. The HyNaC inhibitor, diminazene, slows tentacle movement in *Hydra*. Because *Hydra* express multiple peptide-gated ion channels with a restricted number of FLPs as ligands (35), FLPs may be important for fast transmission at neuro-muscular junction in cnidarians. The function of Hydra-RFamide IV in *Hydra* is unknown.

Highly specialized mechanoreceptor cells, called stinging cells or nematocytes, that are important for capturing prey and defense are present in cnidarians (36). Two- and three-cell synaptic pathways, including synapses between nematocytes and nearby nerve cells, are present in the epidermis of the sea anemone tentacles (37, 38). Cnidarian sensory function is probably mediated by FLPs, as evidenced by anti-FMRFamide and anti-RFamide antibody staining in the tentacles of four classes of cnidaria. Thus, FLPs probably mediate chemosensory regulation of cnidocyte discharge (39). The epidermal sensory cells of the spot ocellus in *Aurelia* are also positive for FMRFamide (40), which may inhibit spontaneous firing of nematocytes.

FLPs also play a key role in cnidarian reproduction, larval movement, and metamorphosis. Reproduction of colonial octocorals such as *Renilla koellikeri* occurs via spawning and exfoliation. Intact gamete follicles are released into the water during spawning. These follicles rupture during exfoliation, releasing the gametes. Antho-RFamide is present in ciliated neurons in the epithelium of follicles of *Renilla koellikeri* and induces exfoliation of the epithelium and subsequent release of the gametes into water (41). Light enhances the potency of Antho-RFamide (41).

The colony-forming marine hydroid, *Hydractinia echinata*, is closely related to freshwater *Hydra*. Fertilized eggs of this species undergo rapid cleavage divisions for about 1 day and develop into spindle-shaped planula larvae in about 3 days (42). Planula larvae are capable of migrating toward light (43), and they metamorphose into adult polyps when they receive appropriate environmental stimuli (44, 45). Hydra-RFamide I inhibits the migration of planula larvae, thus modulating phototaxis by inhibiting myomodulation (43). Metamorphosis is also inhibited by this peptide, leading to the suggestion that the function of endogenous FLPs is to stabilize the larval stage (46). Thus, FLPs may play a role in regulating the movement of planula larvae prior to metamorphosis, possibly linking movement to chemotactic or phototactic processes (47). Sensory neurons that express FLPs are present in planula larvae, suggesting that migration and metamorphosis of these animals may be mediated by secretion of endogenous neuropeptides in response to environmental stimuli.

### GLWamides

GLWamides are characterized by certain features at their N- and C-termini. Most GLWamides have a GLWamide motif at the C-terminus (Table 2). Seven GLWamide peptides are found in *Hydra*, and they include X-Pro or X-Pro-Pro at their N-termini (Table 2) (7, 49). In the anthozoan *Anthopleura elegantissima*, Metamorphosin A (MMA) that is a member of the GLWamide family has an N-terminal pyroglutamine (Table 2) (48). Both N-terminal modifications produce resistance to aminopeptidase (52).

**Table 2:** GLWamide family peptides in cnidarians.

**Name**	**Peptide sequence**	**Species**	**Reference**
MMA	pQQPGLWamide	Anthopleura elegantissima	(48)
Hym-53	NPYPGLWamide	Hydra magnipapillata	(7, 49, 50)
Hym-54	GPMTGLWamide		
Hym-248	EPLPIGLWamide		
Hym-249	KPIPGLWamide		
Hym-331	GPPPGLWamide		
Hym-338	GPP^h^PGLWamide		
Hym-370	KPNAYKGKLPIGLWamide		
Hydra-LWamideVI	RLPLGLWamide		
Hydra-LWamide VIII	pQPPIGMWamide		
He-LWamide I	pQRPPGLWamide	Hydractinia echinata	(51)
He-LWamide II	KPPGLWamide		
Ae-LWamide I	pQQHGLWamide	Actinia equine	(51)
Ae-LWamide II	pQNPGLWamide		
Ae-LWamide III	pQPGLWamide		
Ae-LWamide IV	pQKAGLWamide		
Ae-LWamide V	pQLGLWamide		
Ae-LWamide VI	RSRIGLWamide		
Ae-MWamide	pQDLDIGMWamide		
MMA	pQQPGLWamide		
As-LWamide I	pQQAGLWamide	Anemonia sulcata	(51)
As-LWamide II	pQHPGLWamide		
As-IWamide	pQERIGIWamide		
Ae-LWamide II	pQNPGLWamide		
MMA	pQQPGLWamide		

*pQ, pyroglutamate; ^h^P, hydroxyproline*.

GLWamide cDNAs are found in other cnidarians as well. A cDNA encoding a preprohormone with 11 immature peptide sequences, nine of which are unique, was cloned from *Hydra magnipapillata* (Figure 2B) (50). The corresponding gene includes one copy of Hym-53 (NPYPGLWamide), Hym-54 (GPMTGLWamide), Hym-249 (KPIPGLWamide), and Hym-370 (KPNAYKGKLPIGLWamide); two copies of Hym-248 (EPLPIGLWamide); and t copies of Hym-331 (GPPPGLWamide), as well as two additional putative GLWamides (Hydra-LWamide VI and VIII) (Table 2). Hydra-LWamide VIII is predicted from this cDNA and probably includes GMWamide at the C-terminus (50). A cDNA encoding GLWamides has been cloned from *Hydractinia echinata* (51) and includes one copy of He-LWamide I and 17 copies of He-LWamide II (Table 2). Two unique cDNAs have been cloned from the anthozoans *Actinia equine* and *Anemonia sulcata* (51). The *Actinia* gene includes one copy of MMA, Ae-LWamide IV, Ae-LWamide V, Ae-LWamide VI, and Ae-MWamide; two copies of Ae-LWamide I and Ae-LWamide III; and four copies of Ae-LWamide II (Table 2). In contrast, the *Anemonia* gene has one copy of MMA, Ae-LWamide II, and As-IWamide; two copies of As-LWamide II; and four copies of As-LWamide I (Table 2) (51). The preprohormones of anthozoans but not hydrozoans include MMA. The peptide is probably a prototype of the family (53). Two other peptides that are possibly generated from the preprohormones of *Actinia* and *Anemonia* are likely processed into -GMWamide (Ae-MWamide) and -GIWamide (As-IWamide) at their C-terminus (Table 2). Whether these two peptides and Hydra-LWamide VIII belong to the GLWamide family is uncertain, as substitution of the Leu residue in GLWamide with Met or Ile results in deactivation of contractile activity in the retractor muscle of the anthozoan *Anthopleura fuscoviridis* (54).

The various species of *Hydractinia* generally live on hermit crab shells. The *Hydractinia* life cycle includes a planula larval stage but no medusa stage. After attaching to snail shells, planula larvae undergo MMA-induced metamorphosis and become polyps after about 1 week (48, 55). MMA thus works as a neurohormone to mediate development in addition to its roles as a neurotransmitter and neuromodulator. In *Hydractinia serrata, Hydra* GLWamides also cause polyp development from planula larvae (7, 49). A common GLWamide sequence is required to induce metamorphosis in *Hydractinia*, and the GLWamide terminus and amidation are essential and specific for inducing metamorphosis (56). Substitution of Gly in GLWamide with another common amino acid (except Cys) decreases or completely inhibits potency of the peptide, and substitution of Leu or Trp in GLWamide with another common amino acid (except Cys) partially or completely blocks its potency for muscle contraction in *Anthopleura fuscoviridis* (54). The precise mechanism of how these peptides induce metamorphosis remains to be determined. Bacteria in the environment produce a chemical that can induce larvae to undergo metamorphosis (48). This chemical signal probably affects sensory neurons in the planula larvae that secrete endogenous GLWamides to induce a phenotypic change in the surrounding epithelial cells. *Hydra* lack a larval stage and develop directly into adults from embryos, and thus, how GLWamide peptides function during early development in *Hydra* is unclear.

Motile planula larvae play a role in sexual reproduction in reef-building corals. These larvae undergo complex metamorphosis after adhering to a substrate, and a juvenile coral colony results. In *Acropora*, Hym-248 induces dose-dependent metamorphosis of nearly 100% of planula larvae into polyps (57). However, the effect of Hym-248 on metamorphosis is species-specific (57, 58). A Hym-248-specific receptor appears to exist in *Acropora*. The receptor may serve as a barrier to ensure specification in corals. In *Hydractinia*, the peptide for their receptors is loose. The possible receptors may share certain common sequences and binding sites. Hym-248-related peptide(s) are expected to be identified in *Acropora*.

In *Hydra*, all GLWamide peptides serve as myoactive peptides to activate sphincter muscle contraction and bud detachment (7). The sphincter muscle is involved in bud detachment. To test myoactivity in *Hydra*, nerve-free tissue of epithelial hydra is typically used (59, 60). When normal *Hydra* that contains nerve cells is treated with the peptides, they exhibit the same effect as epithelial *Hydra*. GLWamides are synthesized and expressed in nerve cells (49) and thus function as neurotransmitters or neuromodulators at the neuro-muscular junction. Hym-248, which is a *Hydra* GLWamide, induces both bud detachment and body elongation (49). Muscle tissue in *Hydra* runs perpendicular to the ectodermal and endodermal epithelial cells. Hym-248 may bind to two different types of receptors, one that binds all types of GLWamides and one that specifically binds to Hym-248. Substance P (SP) is a highly conserved member of the tachykinin peptide family that is widely expressed throughout the animal kingdom (61). It binds to tachykinin receptors [neurokinin-1, 2, and 3 receptor (NK1R, NK2R, and NK3R)] that belong to G-protein-coupled receptors (GPCRs). SP preferentially activates NK1R. This difference of specificity against other tachykinin peptides can be accounted for the conformational flexibility of the short and linear peptides and ligand binding affinity for the receptors (62). Probably, the features of both receptors for Hym-248 may depend on the ligand structure and binding affinity for receptors.

All GLWamide family peptides enhance retractor muscle contraction of *Anthopleura* (49). Nerve cells in the sea anemone retractor muscle stain strongly with a GLWamide motif-specific antibody, similar to the nervous system of *Hydra* (49).

In *Hydractinia echinata*, GLWamide and RFamide neuropeptides modulate planula larva migration. He-LWamide II, which is a GLWamide, induces migration by extending the active period (43). GLWamides and FLPs antagonize one another to modulate migration of *Hydractinia echinata* planula larvae.

In hydrozoan jellyfish, maturation of oocytes and spawning are initiated by light-dark cycles in natural conditions within 1 second (63). Exposure to Hym-53 for < 2 min is sufficient for oocyte maturation and spawning (64). Thus, neuropeptides function as hormones that modulate the first step that determines whether oocytes undergo irreversible meiosis after light exposure.

### Hym-176 (APFIFPGPKVamide)

Hym-176 was a newly identified as a neuropeptide (Table 3) (7, 65). The gene that encodes Hym-176 is strongly expressed in the neurons of the lower peduncle and weakly expressed in the gastric region (67). This peptide induces contraction of the ectodermal muscle in *Hydra* (65). This region-specific neuron subset correlates with the myoactivity of the peptide. Hym-176 has no effects on muscle contraction in *Anhtopleura*, metamorphosis in *Hydractinia*, and oocyte maturation and spawning in *Cytaeis*. And also, the gene encoding the peptide (*Hym-176A*) is just isolated from *Hydra* (Figure 2C) (66, 67). Thus, the peptide is species-specific.

**Table 3:** Hym-176, Hym-357, and their related peptides in Hydra.

**Name**	**Peptide sequence**	**Species**	**Reference**
Hym-176	APFIFPGPKVamide	*Hydra magnipapillata*	(65)
Hym-176C	YPFYNQNPKVamide		(66)
Hym-176D	NPKNKNFMIFVGPKVamide		(66)
Hym-357	KPAFLFKGYKPamide	Hydra magnipapillata	(20, 66)
Hym-690	KPLYLFKGYKPamide		(20, 66)

The gene that encodes Hym-176 also encodes a second peptide, Hym-357 (KPAFLFKGYKPamide) (Figure 2C) (Table 3). This neuropeptide was identified in a screen for myoactive peptides (20). Detailed observations suggest that Hym-357 neurons activate other neurons to release neurotransmitters for induction of muscle contraction.

To identify the homologous gene that encodes Hym-176, Noro and coworkers found four candidate genes in the freshwater polyp *Hydra magnipapillata* (66). No authentic Hym-176 is present in the four paralogues (Figure 2C) (66). The cDNAs, *Hym-176C* and *Hym-176D*, encode one copy of a Hym-176-homologous peptide (Figure 2C) (Table 3). Hym-357 is encoded in both the gene that encodes Hym-176 and the gene that encodes Hym-176B (Figure 2C) (66). *Hym-176C* encodes Hym-690 (KPLYLFKGYKPamide), which is closely related to Hym-357 (Figure 2C) (Table 3) (20). *Hym-176E* appears not to have Hym-176- and Hym-357-related peptides (Figure 2C). The function of Hym-176C and D and Hym-690 has not yet been characterized in *Hydra*.

### Hym-355 (FPQSFLPRGamide)

Hym-355 is a member of the PRXamide family of peptides that have PRXamide at their C-terminal region (Figure 2D) (Table 4) (68) and are subdivided into three groups in invertebrates: (a) neuropeptides that induce pheromone biosynthesis (70) and similar molecules, (b) small cardioactive peptides (71–73), and (c) antho-RPamide (52) and similar molecules. Antho-RPamide (LPPGPLPRPamide) is located in neurons of sea anemones and induces tentacle contraction. Thus, the peptide is involved in neurotransmission. PRXamide peptides have been identified in many invertebrates. Hym-355 is homologous to members of sub-group (c), including LPPGPLPRPamide (*Anthopleura elegantissima*) (Table 4), AAPLPRLamide (*Urechis unicinctus*) (74), QPPLPRYamide (*Helix pomatia*), and pQPPLPRYamide (*Helix pomatia*) (75). GPRGGRATEFGPRGamide and GPRGGREVNLEGPRGamide both have PRGamide at their C-termini and are expressed in the sea anemone *Nematostella vectensis* (Table 4) (23). The gene encoding the PRGamides is expressed in neurons (23), indicating that the PRGamides are neuropeptides.

**Table 4:** PRXamide peptides in cnidarians.

**Name**	**Peptide sequence**	**Species**	**Reference**
Hym-355	FPQSFLPRGamide	*Hydra magnipapillata*	(68)
PRGamide (ID:11)	GPRGGRATEFGPRGamide	Nematostella vectensis	(23)
PRGamide (ID:12)	GPRGGREVNLEGPRGamide		
Antho-RPamide	LPPGPLPRPamide	Anthopleura elegantissima	(52)
MIHs	WPRPamide	Clytia hemisphaerica	(69)
	WPRAamide	Cladonema pacificum	
	RPRPamide		
	RPRAamide		
	RPRGamide		
	RPRYamide		

*MIHs, maturation-inducing hormones*.

Oxytocin-vasopressin superfamily peptides are neuropeptides synthesized in the hypothalamus and secreted from the posterior pituitary gland in mammals. Whether cnidarians express oxytocin/vasopressin superfamily peptides remains an open question in the field of comparative physiology of nervous systems. Immunohistochemical staining suggests that oxytocin/vasopressin superfamily peptides exist in the *Hydra* nervous system (76, 77). Morishita and coworkers (78) purified two peptides, Hym-355 and SFLPRGamide, from *Hydra magnipapillata* using HPLC fractionation and immunologic assays. They demonstrated that the antigen for vasopressin-like immunoreactivity is Hym-355 in the *Hydra* nervous system. The C-terminal region of Hym-355 (PRGamide) is identical to that of vasopressin. Neither antibody against the two peptides discriminates one peptide from the other. Thus, Koizumi et al. (79) performed immunohistochemistry with an anti–Hym-355 antibody and demonstrated immunoreactivity in the nerve rings of *Cladonema radiatum* and *Turritopsis nutricula*. However, whether Hym-355 functions as a neurohypophysial hormone is not well-understood.

**Figure 3:**
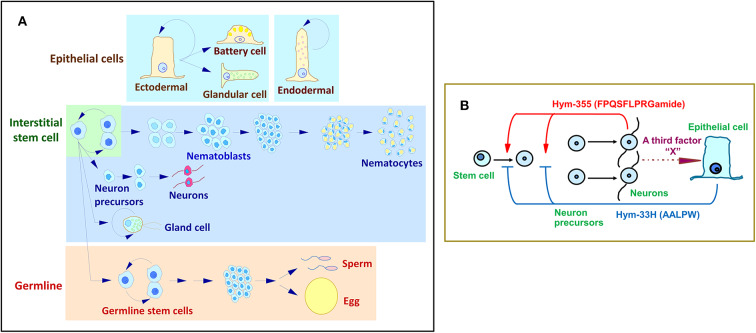
*Hydra* stem cell system. **(A)** Cell differentiation in *Hydra*. **(B)** A feedback model for the control of neuron differentiation that involves the antagonistic action of Hym-355 and the PW peptide, Hym-33H.

The tissue of *Hydra* undergoes continuous renewal (Figure 3A). The number of neurons remains constant. Two groups of peptides, Hym-355 and PW family peptides, regulate this state (7, 68, 80). PW family peptides share the same sequence of Pro-Trp and are identified as epitheliopeptides (81).

Hym-355 increases early neuron differentiation, and Hym-33H (AALPW) blocks neuron differentiation (68, 80). Simultaneous treatment with Hym-355 and Hym-33H results in a normal level of neuron differentiation. Taken together, the observations are consistent with a feedback model that modulates the homeostasis of neuronal differentiation in *Hydra* (Figure 3B) (68). This model suggests that Hym-355, which is synthesized by neurons, enhances early neuronal differentiation. To balance differentiation, epithelial cells produce PW peptides. A third factor termed as X in Figure 3B may control synthesis and secretion of PW family peptides. Hym-355, PW peptides, and the putative third factor may work together to maintain a constant neuronal density in *Hydra*. Hym-355 induces interstitial stem cells to undergo neuron differentiation and also induces retractor muscle contraction in the sea anemone *Anthopleura fuscoviridis* (68).

A member of the GLWamide family, Hym-53 (NPYPGLWamide) (Table 2), and Hym-355 induce oocyte maturation and spawning, but the effect of Hym-53 is stronger than that of Hym-355. Hym-355-like immunoreactivity is observed in neurons in *Cytaeis* (63). Possibly, neurons expressing Hym-53- and Hym-355-like peptides contribute downstream of light receptors in oocyte maturation and spawning in *Cytaeis*. Takeda and coworkers demonstrated that endogenous peptides including W/RPRPamide peptides are involved in oocyte maturation (Table 4) (69). RPRYamide, RPRGamide, WPRAamide, and RPRAamide may act as maturation-inducing hormones (MIHs) (Table 4) (69). Takeda et al. (69) also demonstrated that MIH peptides are synthesized by neurons in the gonad, and probably act on the oocyte surface. They propose that hydrozoan MIHs and neuropeptides are evolutionally linked to regulate reproduction upstream of MIHs in bilaterian species (69).

### FRamide Family

During research aimed at systematic identification of peptide signaling molecules in *Hydra* (7), two novel neuropeptides, FRamide-1 (IPTGTLIFRamide) and FRamide-2 (APGSLLFRamide), were identified (Table 5) (82). Among *Hydra* EST and genome databases (8), we can rapidly identify peptide transcripts and their genes. The two peptides and the single gene encoding both peptides were identified using this exact approach (Figure 2E).

**Table 5:** FRamide family peptides in Hydra.

**Name**	**Peptide sequence**	**Species**	**Reference**
FRamide-1	IPTGTLIFRamide	*Hydra magnipapillata*	(82)
FRamide-2	APGSLLFRamide		

FRamide-1 (IPTGTLIFRamide) and FRamide-2 (APGSLLFRamide) exhibit opposing effects even though they are encoded by the same gene. The former peptide evokes body column elongation due to endodermal muscle contraction, whereas the latter peptide evokes body column contraction due to ectodermal muscle contraction (82). Two explanations for these seemingly contradictory observations are possible. One possibility is that the release of each peptide is differentially regulated (83, 84), and the other possibility is that each peptide is processed in a different type of neuron (85). Additionally, the opposing effects of FRamide family peptides may be ligand binding affinity for one receptor (62). In higher animals, most neuropeptides bind to GPCRs that are localized at the target cell. To understand the opposite effects, identification of FRamide-specific receptors on the target cells is important.

## Conclusion

**Figure 4:**
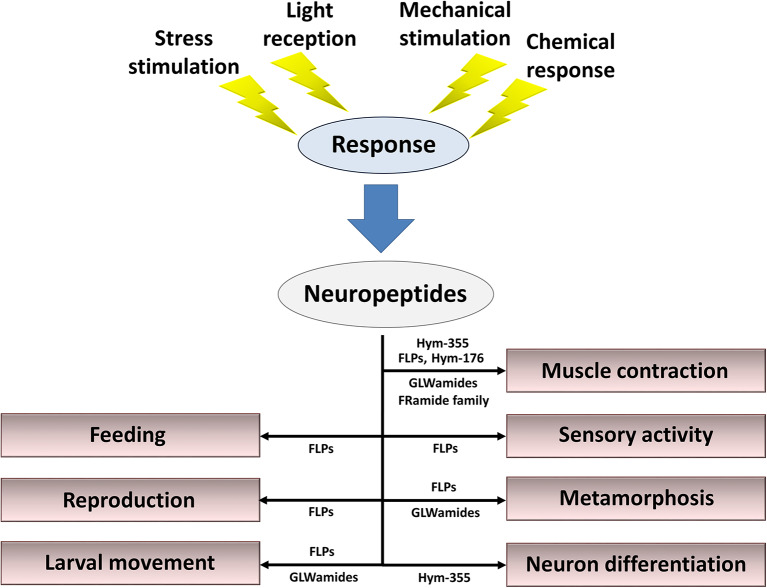
Summary of roles for neuropeptides in the control of behavior, reproduction, metamorphosis, and tissue maintenance. Cnidarian peptide signaling molecules function together and/or separately to maintain the organism's lifestyle in response to stress stimulation, light reception, mechanical stimulation, and chemical stimulation.

Neuropeptides released from nerve cells in response to a variety of stimuli are mandatory for fine-tuned regulation of behavior, reproduction, metamorphosis, and tissue maintenance (Figure 4). Here, I described 57 types of neuropeptides so far identified in cnidarians. However, the study of neuropeptides is still in its infancy. Additional novel peptides will likely be found (86), including neuropeptides, thus enabling elucidation of the mechanisms that regulate the physiology and development of cnidarians and increasing our understanding of peptide function in other species.

It is important to elucidate functional interaction between neuropeptides and receptors for the verification of their biological roles and evolutionary processes. However, no receptors for the neuropeptides remain to be identified in *Hydra* and cnidarians. Recently, Shiraishi and coworkers developed the machine-learning-assisted strategy for the identification of novel peptide–receptor pairs (87). As they indicate the multiplicity of use of the strategy, it is worth to use the strategy for increasing the receptor (especially GPCR) repertoire as many as possible on *Hydra* and cnidarians. When neuropeptide-GPCR pairs are efficiently and systematically elucidated in a phylogenetically critical Hydrozoa *Hydra magnipapillata, Hydra* provides cnidarian perspectives into evolution of GPCRs.

The cells of *Hydra* are well-characterized and belong to the epithelial cell lineage and the interstitial stem cell lineage (Figure 3A). However, knowledge of the molecules and biochemical mechanisms of the cells remains limited. The single-cell RNA sequencing technique sheds light on the complete molecular diversity of the cells in *Hydra*. Siebert and coworkers (88) applied this approach to the homeostatic adult *Hydra*. They drew a molecular map of the *Hydra* nervous system and unlocked the door toward understanding the molecular basis of morphogenesis and regeneration in *Hydra*.

## Author Contributions

TT wrote the original review manuscript draft.

## Conflict of Interest

The author declares that the research was conducted in the absence of any commercial or financial relationships that could be construed as a potential conflict of interest.

## References

[1] Galliot B Quiquand M Ghila L de Rosa R Miljkovic-Licina M Chera S. Origins of neurogenesis, a cnidarian view. Dev. Biol. (2009) 332:2–24. 10.1016/j.ydbio.2009.05.563 19465018

[2] David CN Gierer A. A cell cycle kinetics and development of *Hydra attenuate*. III. Nerve and nematocyte differentiation. J Cell Sci. (1974) 16:3375. 4448826 10.1242/jcs.16.2.359

[3] Schmidt T David CN. Gland cells in *Hydra*: Cell cycle kinetics and development. J Cell Sci. (1986) 85:197–215. 3539952 10.1242/jcs.85.1.197

[4] Bosch TCG David CN Stem cells of *Hydra magnipapillata* can differentiate intosomatic cells and germ line cells. Dev Biol. (1987) 121:182–91. 10.1016/0012-1606(87)90151-5

[5] Grimmelikhuijzen CJP Leviev I Carstensen K. Peptides in the nervous system of cnidrians: structure, function and biosynthesis. Int Rev Cytol. (1996) 167:37–89. 10.1016/S0074-7696(08)61345-5 8768492

[6] Kass-Simon G Pierobon P. Cnidarian chemical neurotransmission, an updated overview. Comp Biochem Physiol A Mol Integr Physiol. (2007) 146:9–25. 10.1016/j.cbpa.2006.09.008 17101286

[7] Takahashi T Muneoka Y Lohmann Y deHaro MSL Solleder G Bosch TCG. Systematic isolation of peptide signaling molecues regulating development in hydra: LWamide and PW families. Proc Natl Acad Sci USA. (1997) 94:1241–6. 10.1073/pnas.94.4.1241 9037037 PMC19775

[8] Chapman JA Kirkness EF Simakov O Hampson SE Mitros T Weinmaier T. The dynamic genome of *Hydra*. Nature. (2010) 464:592–6. 10.1038/nature08830 20228792 PMC4479502

[9] Price DA Greenberg MJ Structure of a molluscan cardioexcitatory neuropeptide. Science. (1977) 97:670–1. 10.1126/science.877582 877582

[10] Price DA Greenberg MJ. Purification and characterization of a cadioexcitatory neuropeptide from the central ganglia of a bivalve mollusk. Prep Biochem. (1977) 7:261–81. 10.1080/00327487708061643 909875

[11] Price DA Greenberg MJ The hunting of the FaRPs: The distribution of FMRFamide-related peptides. Biol Bull. (1989) 177:198–205. 10.2307/1541933

[12] Espinoza E Carrigan M Thomas SG Shaw G Edison AS. A statistical view of FMRFamide neuropeptide diversity. Mol Neurobiol. (2000) 21:35–56. 10.1385/MN:21:1-2:035 11327149

[13] Krajniak KG. Invertebrate FMRFamide related peptides *Protein Pept Lett*. (2013) 20:647–70. 10.2174/0929866511320060005 22630125

[14] Grimmelikhuijzen CJP Graff D. Isolation of pyroGlu-Gly-Arg-Phe-NH2 (Antho-RFamide), a neuropeptide from sea anemones. Proc Natl Acad Sci USA. (1986) 83:9817–21. 10.1073/pnas.83.24.9817 2879288 PMC387233

[15] Moosler A Rhinehart KL Grimmelikhuijzen CJP. Isolation of three novel peptides, the Cyanea-RFamides I-III, from scyphomedusae. Biochem Biophys Res Commun. (1997) 236:743–9. 10.1006/bbrc.1997.7022 9245726

[16] Grimmelikhuijzen CJP Hahn M Rhinehart KL Spencer AN. Isolation of pyroGlu-Leu-Leu-Gly-Gly-Arg-Phe-NH_2_ (Pol-RFamide), a novel neuropeptide from hydromedusae. Brain Res. (1988) 475:198–203. 10.1016/0006-8993(88)90219-3 2905621

[17] Grimmelikhuijzen CJP Rhinehart KL Spencer AN. Isolation of the neuropeptide less than Glu-Trp-Leu-Lys-Gly-Arg-Phe-NH_2_ (Pol-RFamide II) from the hydromedusa *Polyorchis penicillatus*. Biochem Biophys Res Commun. (1992) 183:375–82. 10.1016/0006-291X(92)90491-3 1550547

[18] Moosler A Rhinehart KL Grimmelikhuijzen CJP. Isolation of four novel neuropeptides, the Hydra-RFamides I-IV, from *Hydra magnipapillata*. Biochem Biophys Res Commun. (1996) 229:596–602. 10.1006/bbrc.1996.1849 8954943

[19] Darmer D Hauser F Nothacker HP Bosch TCG Williamson M Grimmelikhuijzen CJP. Three different prohormones yield a variety of Hydra-RFamide (Arg-Phe-NH_2_) neuropeptide in *Hydra magnipapillata*. Biochem J. (1998) 332:403–12. 10.1042/bj3320403 9601069 PMC1219495

[20] Fujisawa T. *Hydra* peptide project 1993–2007. Dev Growth Differ. (2008) 50:S257–68. 10.1111/j.1440-169X.2008.00997.x 18459981

[21] Gajewski M Schmutzler C Plickert G. Structure of neuropeptide precursors in cnidarian. Ann N Y Acad Sci. (1998) 839:311–5. 10.1111/j.1749-6632.1998.tb10782.x 9629167

[22] Anctil M. Chemical transmission in the sea anemone *Nematostella vectensis*: a genomic perspective. Comp Biochem Physiol D. (2009) 4:268–89. 10.1016/j.cbd.2009.07.001 20403752

[23] Hayakawa E Watanabe H Menschaert G Holstein TW Baggerman G Schoofs L. A combined strategy of neuropeptide prediction and tandem mass spectrometry identifies evolutionarily conserved ancient neuropeptides in the sea anemone *Nematostella vectensis*. PLoS ONE. (2019) 14:e0215185. 10.1371/journal.pone.0215185 31545805 PMC6756747

[24] Darmer D Schmutzler C Diekhoff D Grimmelikhuijzen CJP. Primary structure of the precursor for the sea anemone neuropeptide Antho-RFamide (< Glu-Gly-Arg-Phe-NH_2_). Proc Natl Acad Sci USA. (1991) 88:2555–9. 10.1073/pnas.88.6.2555 1706527 PMC51271

[25] Schmutzler C Darmer D Diekhoff D Grimmelikhuijzen CJP. Identification of a novel type of processing sites in the precursor for the sea anemone neuropeptide Antho-RFamide (< Glu-Gly-Arg-Phe-NH_2_) from *Anthopleura elegantissima*. J Biol Chem. (1992) 267:22534–41. 1429603

[26] Reinscheid RK Grimmelikhuijzen CJP. Primary structure of the precursor for the anthozoan neuropeptide Antho-RFamide from *Renilla kollikeri*: evidence for unusual processing enzymes. J Neurochem. (1994) 62:1214–22. 10.1046/j.1471-4159.1994.62031214.x 7906718

[27] Schmutzler C Diekhoff D Grimmelikhuijzen CJP. The primary structure of the Pol-RFamide neuropeptide precursor protein from the hydromedusa *Polyorchis penicillatus* indicates a novel processing proteinase activity. Biochem J. (1994) 299:431–6. 10.1042/bj2990431 7909659 PMC1138290

[28] McFarlane ID Graff D Grimmelikhuijzen CJP Excitatory actions of Antho-RFamide, an anthozoan neuropeptide, on muscles and conducting systems in the sea anemone. Calliactis parasitica. J Exp Biol. (1987) 133:157–68.

[29] Anctil M Grimmelikhuijzen CJP. Excitatory action of the native neuropeptide Antho-RFamide in muscles in the pennatulid *Renilla kollikeri*. Gen Pharmacol. (1989) 20:381–4. 10.1016/0306-3623(89)90277-2 2568307

[30] Shimizu H Fujisawa T. Peduncle of *Hydra* and the heart of higher organisms share a common ancestral origin. Genesis. (2003) 36:182–6. 10.1002/gene.10213 12929088

[31] Cottrell GA Green KA Davis NW. The neuropeptide Phe-Met-Arg-Phe-NH_2_(FMRFamide) can activate a ligand-gated ion channel in *Helix* neurons. Pflugers Arch. (1990) 416:612–4. 10.1007/BF00382698 1700364

[32] Lingueglia E Champigny G Lazdunski M Barbry P. Cloning of the amiloride-sensitive FMRFamide peptide-gated sodium channel. Nature. (1995) 378:730–3. 10.1038/378730a0 7501021

[33] Golubovic A Kuhn A Williamson M Kalbacher H Holstein TW Grimmelikhuijzen CJP. A peptide-gated ion channel from the freshwater polyp *Hydra*. J Biol Chem. (2007) 282:35098–103. 10.1074/jbc.M706849200 17911098

[34] Dürrnagel S Kuhn A Tsiairis CD Williamson M Kalbacher H Grimmelikhuijzen CJP. Three homologous subunits form a high affinity peptide-gated ion channel in *Hydra*. J Biol Chem. (2010) 285:11958–65. 10.1074/jbc.M109.059998 20159980 PMC2852933

[35] Assmann M Kuhn A Dürrnagel S Holstein TW Gründer S. The comprehensive analysis of DEG/ENaC subunits in *Hydra* reveals a large variety of peptide-gated channels, potentially involved in neuromuscular transmission. BMC Biol. (2014) 12:84. 10.1186/s12915-014-0084-2 25312679 PMC4212090

[36] Tardent P The cnidarian cnidocyte, a high tech cellular weaponry. BioEssays. (1995) 17:351–62. 10.1002/bies.950170411

[37] Holtmann M Thurm U. Mono- and oligo-vesicular synapses and their connectivity in a Cnidarian sensory epithelium (*Coryne tubulosa*). J Comp Neurol. (2001) 432:537–49. 10.1002/cne.1118 11268013

[38] Westfall JA Elliott CF Carlin RW. Ultrastructural evidence for two-cell and three-cell neural pathways in the tentacle epidermis of the sea anemone *Aiptasia pallida*. J Morphol. (2002) 251:83–92. 10.1002/jmor.1075 11746469

[39] Anderson PA Thompson LF Moneypenny CG. Evidence for a common pattern of peptidergic innavations of cnidocytes. Biol Bull. (2004) 207:141–6. 10.2307/1543588 15501855

[40] Nakanishi N Hartenstein V Jacobs DK. Development of the rhopalial nervous system in *Aurelia* sp. 1 (Cnidaria, Scyphozoa). Dev Genes Evol. (2009) 219:301–17. 10.1007/s00427-009-0291-y 19543911 PMC2706374

[41] Tremblay ME Henry J Anctil M. Spawning and gamete follicle rupture in the cnidarian *Renilla Koellikeri*: effects of putative neurohormones. Gen Comp Endocrinol. (2004) 137:9–18. 10.1016/j.ygcen.2004.02.009 15094331

[42] Plickert G Kroiher M Munck A. Cell proliferation and early differentiation during embryonic development and metamorphosis of *Hydractinia echinata*. Development. (1988) 103:795–803. 2907879 10.1242/dev.103.4.795

[43] Katsukura Y Ando H David CN Grimmelikhuijzen CJP Sugiyama T. Control of planula migration by LWamide and RFamide neuropeptides in *Hydractinia echinata*. J Exp Biol. (2004) 207:1803–10. 10.1242/jeb.00974 15107436

[44] Leitz T. Metamorphosin A and related compounds: a novel family of neuropeptides with morphogenetic activity. Ann N Y Acad Sci. (1998) 839:105–10. 10.1111/j.1749-6632.1998.tb10740.x28306026

[45] Leitz T Induction of metamorphosis of the marine hydrozoan *Hydractinia echnata* Fleming, 1828. Biofouling. (1998) 12:173–87. 10.1080/08927019809378353

[46] Katsukura Y David CN Grimmelikhuijzen CJP Sugiyama T. Inhibition of metamorphosis by RFamide neuropeptides in planula larvae of *Hydractinia echinata*. Dev Genes Evol. (2003) 213:579–86. 10.1007/s00427-003-0361-5 14586653

[47] Seipp S Schmich J Will B Schetter Plickert G Leitz T. Neuronal cell death during metamorphosis of *Hydractinia echinata*. (Cnidaria, Hydrozoa). Invertebr Neurosci. (2010) 10:77–91. 10.1007/s10158-010-0109-7 21104287

[48] Leitz T Morand K Mann M. Metamorphosin A: a novel peptide controlling development of the lower metazoan *Hydractinia echinata* (Coelenterata, Hydrozoa). Dev Biol. (1994) 163:440–6. 10.1006/dbio.1994.1160 7911112

[49] Takahashi T Kobayakawa Y Muneoka Y Fujisawa Y Mohri S Hatta M. Identification of a new member of the GLWamide peptide family: physiological activity and cellular localization in cnidarian polyps. Comp Biochem Physiol Part B. (2003) 135:309–24. 10.1016/S1096-4959(03)00088-5 12798941

[50] Leviev I Williamson M Grimmelikhuijzen CJP. Molecular cloning of a preprohormone from *Hydra magnipapillata* containing multiple copies of Hydra-LWamide (Leu-Trp-NH_2_) neuropeptides: evidence for processing at Ser and Asn residues. J Neurochem. (1997) 68:1319–25. 10.1046/j.1471-4159.1997.68031319.x 9048780

[51] Gajewski M Leitz T Schlosherr J Plickert G. LWamides from cnidaria constitute a novel family of neuropeptides with morphogenetic activity. Roux's Arch Dev Biol. (1996) 205:232–42. 10.1007/BF00365801 28306026

[52] Carstensen K Rinehart KL McFarlane ID Graff D Grimmelikhuijzen CJP. Isolation of Leu-Pro-Pro-Gly-Pro-Leu-Pro-Arg-Pro-NH_2_ (Antho-RPamide), and N-terminally protected, biologically active neuropeptide from sea anemones. Peptides. (1992) 13:851–7. 10.1016/0196-9781(92)90040-A 1480510

[53] Leitz T Lay M. Metamorphosin A is a neuropeptide. Roux's Arch Dev Biol. (1995) 204:276–9. 10.1007/BF00208495 28306123

[54] Takahashi T Ohtani M Muneoka Y Aimoto S Hatta M Shimizu H Structure-activity relation of LWamide peptides synthesized with a multipeptide synthesizer. In: Kitada C, editor. Peptide Chemistry. Osaka: Protein Research Foundation (1997). p. 193–6.

[55] Takahashi T Hatta M. The importance of GLWamide neuropeptides in Cnidarian development and physiology. J Amino Acids. (2011) 2011:1–8. 10.4061/2011/424501 22312460 PMC3268022

[56] Schmich J Trepel S Leitz T. The role of GLWamides in metamorphosis of *Hydractinia echinata*. Dev Genes Evol. (1998) 208:267–73. 10.1007/s004270050181 9683742

[57] Iwao K Fujisawa T Hatta M A cnidarian neuropeptide of the GLWamide family indices metamorphosis of reef-building corals in the genus *Acropora*. Coral Reefs. (2002) 21:127–9. 10.1007/s00338-002-0219-8

[58] Erwin PM Szmant AM Settlement induction of Acropora palmate planulae by a GLW-amide neuropeptide. Coral Reefs. (2010) 29:929–39. 10.1007/s00338-010-0634-1

[59] Marcum BA Campbell RD. Development of *hydra*. lacking nerve and intestinal cells. J Cell Sci. (1978) 29:17–33. 627604 10.1242/jcs.29.1.17

[60] Campbell RD. Elimination of *Hydra*. intestinal and nerve cells by means of colchicines. J Cell Sci. (1976) 21:1–13. 932105 10.1242/jcs.21.1.1

[61] Zieglgänsberger W. Substance P and pain chronicity. Cell Tissue Res. (2019) 375:227–41. 10.1007/s00441-018-2922-y 30284083 PMC6335504

[62] Ganjiwale A Cowsik SM. Molecular recognition of tachykinin receptor selective agonists: insights from structural studies. Mini Rev Med Chem. (2013) 13:2036–46. 10.2174/13895575113139990079 23937231

[63] Takeda N Kyozuka K Deguchi R. Increase in intracellular cAMP is a prerequisite signal for initiation of physiological oocyte meiosis maturation in the hydrozoan *Cytaeis uchidae*. Dev Biol. (2006) 298:248–58. 10.1016/j.ydbio.2006.06.034 16884710

[64] Takeda N Nakajima Y Koizumi O Fujisawa T Takahashi T Matsumoto M. Neuropeptides trigger oocyte maturation and subsequent spawning in the hydrozoan jellyfish *Cytaeis uhcidae*. Mol Reprod Dev. (2013) 80:223–232. 10.1002/mrd.22154 23341254

[65] Yum S Takahashi T Koizumi O Ariura Y Kobayakawa Y Mohri S. A novel neuropeptide, Hym-176, induces contraction of the ectodermal muscle in *Hydra magnipapillata*. Biochem Biophys Res Commun. (1998) 248:584–90. 10.1006/bbrc.1998.8831 9703970

[66] Noro Y Yum S Fujisawa-Nishimiya C Busse C Shimizu H Mineta K. Regionalized nervous system in *Hydra* and the mechanism of its development. Gene Expr Patterns. (2019) 31:42–59. 10.1016/j.gep.2019.01.003 30677493

[67] Yum S Takahashi T Hatta M Fujisawa T. The structure and expression of a preprohormone of a neuropeptide, Hym-176 in *Hydra magnipapillata*. FEBS Lett. (1998) 439:31–4. 10.1016/S0014-5793(98)01314-3 9849871

[68] Takahashi T Koizumi O Ariura Y Romanovitch A Bosch TCG Kobayakawa Y. A novel neuropeptide, Hym-355, positively regulates neuron differentiation in *Hydra*. Development. (2000) 127:997–1005. 10.1016/S1095-6433(99)90367-7 10662639

[69] Takeda N Kon Y Artigas GQ Lapebie P Barreau C Koizumi O. Identification of jellyfish neuropeptides that act directly as oocyte maturation-inducing hormones. Development. (2018) 145:dev156786. 10.1242/dev.156786 29358214

[70] Raina AK Jaffe H Kempe TG Keim P Blacher RW Fales HM. Identification of a neuropeptide hormone that regulates sex pheromone production in female moths. Science. (1989) 244:796–8. 10.1126/science.244.4906.796 17802237

[71] Muneoka Y Takahashi T Kobayashi M Ikeda T Minakata H Nomoto K. Phylogenetic aspects of structure and action of molluscan neuropeptides. In: Davey KG, Peter RE, Tobe SS, editors. Perspectives in Comparative Endocrinology. Toronto, ON: National Research Council of Canada (1994). p. 109–18.

[72] Morris HR Panico M Karplus A Lloyd PE Piniker B. Identification by FAB-MS of the structure of a new cardioactive peptide from *Aplysia*. Nature. (1982) 300:643–5. 10.1038/300643a0 7144915

[73] Lloyd PE Kupfermann I Weiss KR. Sequence of small cardioactive peptide A: a second member of a class of neuropeptides in *Aplysia*. Peptides. (1987) 8:179–83. 10.1016/0196-9781(87)90184-7 3575150

[74] Ikeda T Kubota I Miki W Nose T Takao T Shimonishi Y Structures and actions of 20 novel neuropeptides isolated from the ventral nerve cords of an echiuroid worm, *Urechis unicinctus*. In: Yanaihara N, editor. Peptide Chemistry 1992. Leiden: Protein Research Foundation (1993). p. 583–5. 10.1007/978-94-011-1474-5_168

[75] Minakata H Ikeda T Fujita T Kiss T Hiripi L Muneoka Y Neuropeptides isolated from *Helix pomatia*. Part 2. FMRFamide-related peptides, S-Iamide peptides, FR peptides and others. In: Yanaihara N, editor. Peptide Chemistry 1992. Leiden: Protein Research Foundation (1993) 579–82. 10.1007/978-94-011-1474-5_167

[76] Grimmelikhuijzen CJP Dierickx K Boer GJ. Oxytocin/vasopressin-like immunoreactivity is present in the nervous system of hydra. Neuroscience. (1982) 7:3191–9. 10.1016/0306-4522(82)90241-X 6761600

[77] Koizumi O Bode HR. Plasticity in the nervous system of adult hydra. III. Conversion of neurons to expression of a vasopressin-like immunoreactivity depends on axial location. J Neurosci. (1991) 11:2011–20. 10.1523/JNEUROSCI.11-07-02011.1991 2066772 PMC6575488

[78] Morishita F Nitagai Y Furukawa Y Matsushima O Takahashi T Hatta M. Identification of a vasopressin-like immunoreactive substance in hydra. Peptides. (2003)24:17–26. 10.1016/S0196-9781(02)00272-3 12576081

[79] Koizumi O Hamada S Minobe S Hamaguchi-Hamada K Kurumata-Shigeto M Nakamura M. The nerve ring in cnidarians: its presence and structure in hydrozoan medusa. Zoology. (2015) 118:79–88. 10.1016/j.zool.2014.10.001 25498132

[80] Takahashi T Koizumi O Hayakawa E Minobe S Suetsugu R Kobayakawa Y. Further characterization of the PW peptide family that inhibits neuron differentiation in *Hydra*. Dev Genes Evol. (2009) 219:119–29. 10.1007/s00427-009-0272-1 19184097

[81] Takahashi T. Neuropeptides and epitheliopeptides: structural and functional diversity in an ancestral metazoan *Hydra*. Protein Pept Lett. (2013) 20:671–80. 10.2174/0929866511320060006 23030717

[82] Hayakawa E Takahashi T Nishimiya-Fujisawa C Fujisawa T. A novel neuropeptide (FRamide) family identified by a peptidomic approach in *Hydra magnipapillata*. FEBS J. (2007) 274:5438–48. 10.1111/j.1742-4658.2007.06071.x 17894820

[83] Eipper B Mains RE. Structure and biosynthesis of proadrenocorticotropin/endorphin and released peptides. Endocr Rev. (1980) 1:1–27. 10.1210/edrv-1-1-1 6262069

[84] Rosa PA Policastro P Herbert E. A cellular basis for the differences in regulation of synthesis and secretion of ACTH/endorphin peptides in anterior and intermediate lobes of the pituitary. J Exp Biol. (1980) 89:215–37. 6259270 10.1242/jeb.89.1.215

[85] Klumperman J Spijker S van Minnen J Sharp-Baker H Smit AB Geraerts WPM. Cell type-specific sorting of neuropeptides: a mechanism to modulate peptide composition of large-dense-core vesicles. J Neurosci. (1996) 16:7930–40. 10.1523/JNEUROSCI.16-24-07930.1996 8987821 PMC6579229

[86] Koch TL Grimmelikhuijzen CJP. Global neuropeptide annotations from the genomes and transcriptomes of Cubozoa, Scyphozoa, Staurozoa (Cnidaria: Medusozoa), and Octocorallia (Chidaria: Anthozoa). Front. Endocrinol. (2019) 10:831. 10.3389/fendo.2019.00831 31866941 PMC6909153

[87] Shiraishi A Okuda T Miyasaka N Osugi T Okuno Y Inoue J. Repertoires of G protein-coupled receptors for *Ciona*-specific neuropeptides. Proc. Natl. Acad. Sci. USA. (2019) 116:7847–56. 10.1073/pnas.1816640116 30936317 PMC6475428

[88] Siebert S Farrell JA Cazet JF Abeykoon Y Primack AS Schnitzler CE. Stem cell differentiation trajectories in *Hydra* resolved at single-cell resolution. Science. (2019) 365:eaav9314. 10.1126/science.aav9314 31346039 PMC7104783

